# Case Report: Rapid Desensitization to Ocrelizumab for Multiple Sclerosis Is Effective and Safe

**DOI:** 10.3389/fimmu.2022.840238

**Published:** 2022-02-09

**Authors:** Marcelo Vivolo Aun, Fernando Freua, Victor Hugo Rocha Marussi, Pedro Giavina-Bianchi

**Affiliations:** ^1^ Faculdade Israelita de Ciências da Saúde Albert Einstein School of Medicine, São Paulo, Brazil; ^2^ Clinical Immunology and Allergy Division, University of Sao Paulo School of Medicine, São Paulo, Brazil; ^3^ Department of Neurology, Hospital Beneficência Portuguesa de São Paulo, São Paulo, Brazil; ^4^ Neurology Division, University of Sao Paulo School of Medicine, São Paulo, Brazil

**Keywords:** hypersensitivity, allergy, multiple sclerosis, monoclonal antibodies, Ocrelizumab, desensitization

## Abstract

Monoclonal antibodies have become a mainstay of treatment for many inflammatory diseases and malignancies. Multiple sclerosis is a chronic inflammatory, demyelinating, and neurodegenerative disease of the central nervous system and a common cause of disability in young adults. Ocrelizumab is a recombinant humanized monoclonal antibody that targets CD20-positive B cells and has been approved in the treatment of multiple sclerosis. Although considered safe, more than 30% of patients treated with Ocrelizumab developed infusion-related reactions, mostly regarded as mild. When severe, they can lead to a definite suspension of that drug. We present a case report of Ocrelizumab desensitization in a female patient who presented an immediate hypersensitivity reaction (urticaria and angioedema) during the first Ocrelizumab infusion. Although mechanisms involved in the response were not elucidated, the procedure occurred uneventfully and permitted first-line multiple sclerosis treatment maintenances. Desensitization should be considered a safe therapeutic option in patients with immediate hypersensitivity reactions to Ocrelizumab.

## Introduction

Monoclonal antibodies (mAbs) have become a mainstay for many inflammatory diseases and malignancies. There are four different types of mAbs used in the treatment of human disease, listed in decreasing order of immunogenicity: chimeric (suffix “ximab”; e.g., infliximab); humanized (suffix “zumab”; e.g., omalizumab); fully human (suffix “umab”; e.g., adalimumab); and receptor fusion (suffix “cept”; e.g., etanercept) (1, 2). As they are “non-self” proteins, the immune system can recognize these biological products and trigger an immune response (1). Initial biologics included more significant parts of non-human proteins (as chimeric, for example) and more immunogenic. As they became more similar to human proteins, their immunogenicity diminished progressively (1).

Multiple sclerosis (MS) is a chronic inflammatory, demyelinating, and neurodegenerative disease of the central nervous system and a common cause of disability in young adults. MS can be categorized as relapsing (RMS) or primary progressive (PPMS) but is primarily considered a progressive disease in most patients (3).

MS was long thought to be a T-cell-mediated autoimmune disorder, causing inflammatory demyelination and neuronal damage, which slows or prevents nerve signaling (4). More recently, B cells have been shown to play an essential role in the pathogenesis of MS *via* many mechanisms, such as the presentation of autoantigens and costimulatory signals to activate T cells and the secretion of pro-inflammatory cytokines (5).

Ocrelizumab is a recombinant humanized mAb that targets CD20-positive B cells and has been approved for the treatment of RMS and PPMS (1, 4). The precise mechanisms by which Ocrelizumab exerts its therapeutic clinical effects in MS are not fully elucidated. Still, it is believed that it eliminates B cells from the peripheral blood, primarily through antibody-dependent cellular cytotoxicity and to a lesser extent by antibody-dependent cellular phagocytosis, complement-dependent cytotoxicity, and the direct apoptosis of B cells (6).

Ocrelizumab is the first CD20+ B-cell-selective monoclonal antibody for treating MS, at a dose of 600 mg IV twice yearly, with significant benefit on disability progression and with sustained efficacy with continuous efficacy therapy up to 6.5 years in the open-label extensions of the phase III studies (3).

Although considered safe, more than 30% of patients treated with Ocrelizumab in phase III trials developed infusion-related reactions (IRRs), mostly regarded as mild. When IRRs are moderate to severe, they can lead to a definitive suspension of that drug and to a scheme modification, which has been previously described in clinical trials (7).

Rapid drug desensitization (RDD) is a cornerstone in the management of immediate hypersensitivity reactions (IHRs) and can be applied to allergic (IgE-mediated) and non-allergic reactions. It is indicated when there is no alternative drug to replace the one that elicits the initial reaction (8).

We describe a female patient with MS who presented an IHR to Ocrelizumab at the first infusion and was successfully and safely desensitized to that drug.

## Case Description

A 39-year-old woman with a history of progressive left spasticity since the age of 24, extensively investigated by an orthopedist, with no detailed record of fluctuations in the motor condition, was evaluated in a neurological consultation that showed a left pyramidal syndrome with no changes in superficial and deep sensitivity.

**Figure 1:**
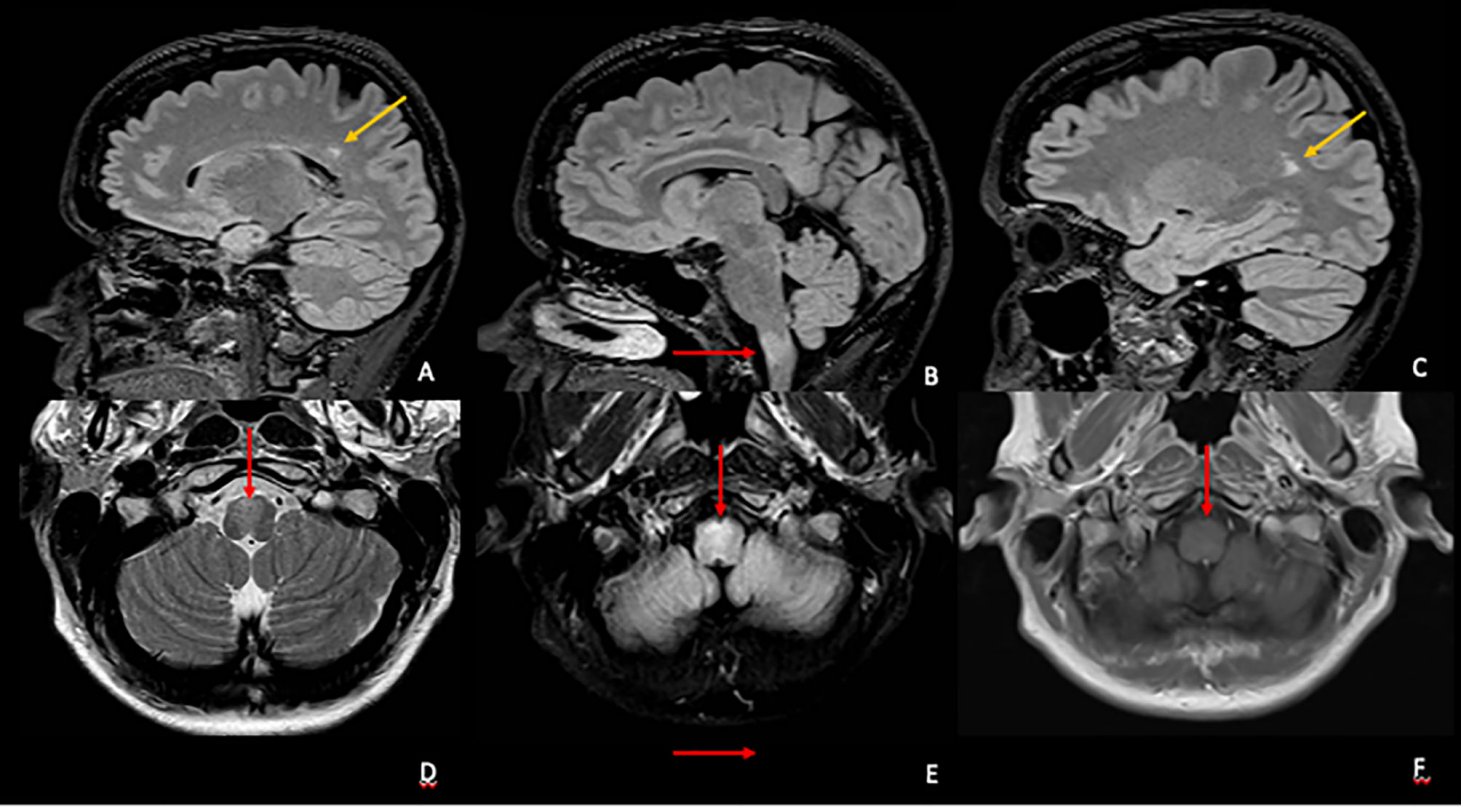
Top row **(A–C)** Sagittal volumetric fluid-attenuated inversion recovery (FLAIR) images showing at least two periventricular hyperintense lesions (yellow arrows) with perivenular distribution and one ventral medula oblongata lesion (red arrow). Bottom row—Axial T2 **(D)**, FLAIR **(E)**, and T1 pos gadolinium **(F)** images showing the ventral medulla oblongata hyperintense lesion compromising pyramidal decussation **(D, E)** without gadolinium enhancement **(F)**.

She was investigated with serological tests of autoimmunity, including serum anti-AQP4 and anti-MOG, with negative results, and a brain, cervical, and thoracic magnetic resonance was performed that showed a bulbar lesion with a demyelinating pattern (**Figure 1**). Given the characteristics of the lesion and the clinical history, the investigation was complemented with a study of the cerebrospinal fluid (average cell count, normal protein levels, and absence of oligoclonal bands) and genetic panel for genetically determined leukoencephalopathies including the *GFAP* gene, to rule out the adult form of Alexander disease. After these last exams, the diagnosis of PPMS was defined, followed by infusion of Ocrelizumab as the only approved disease-modifying therapy for this condition.

**Figure 2:**
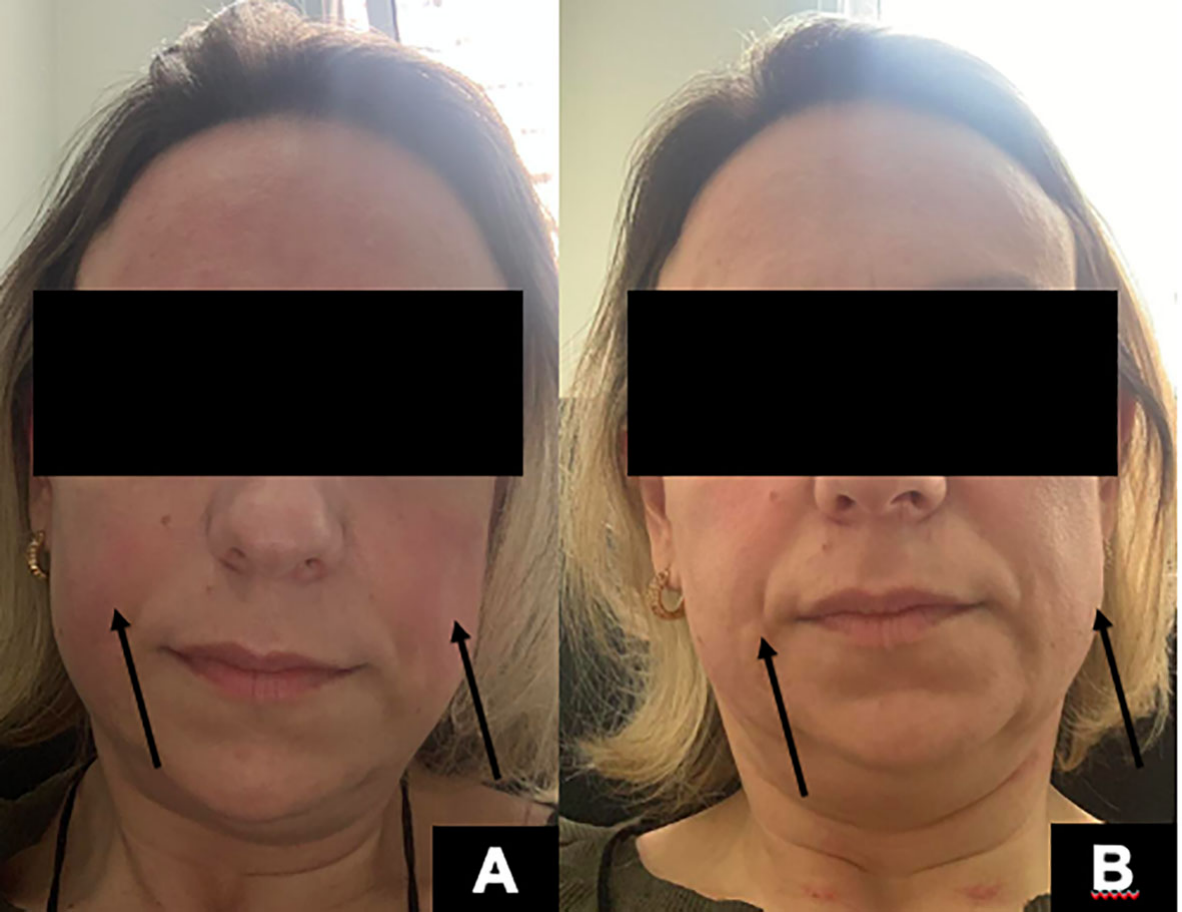
**(A)** Facial erythema and flushing (arrows) during Ocrelizumab infusion. **(B)** Facial angioedema (arrows) minutes after the initial facial erythema.

As defined by clinical trials, the first dose should be 600 mg divided into two 300-mg doses, separated by 14 days, with a premedication scheme including a 100-mg dose of IV methylprednisolone. When about 290 mg of Ocrelizumab had been administered, the patient started to present pruritus and flushing (**Figure 2A**), followed by generalized urticaria and facial angioedema (**Figure 2B**). She did not develop dyspnea, tachycardia, or hypotension. The infusion was stopped, and the reaction was successfully treated with an extra dose of 100 mg methylprednisolone succinate and diphenhydramine 50 mg IV. The patient had a previous history of allergic rhinitis and nonsteroidal anti-inflammatory drugs (NSAIDs)-induced urticaria and angioedema. However, she had never presented any reactions to injectable medications. She had presented three urticaria or angioedema attacks after taking aspirin, ibuprofen, and dipyrone orally.

To maintain MS first-line treatment, she was evaluated by the Allergy Unit, and an RDD was indicated until a therapeutic 300-mg dose as previously described (9). The woman underwent risk stratification and was classified as low risk (Brown Classification grade I IHR with no respiratory or cardiovascular comorbidities) (10). Although it would help define the mechanisms involved and completely stratify the patient’s risk, we could not perform skin tests.

Then, the patient was submitted to the 3-bag, 12-step RDD protocol (9), which is summarized in **Table 1**. She was premedicated with 100 mg methylprednisolone and diphenhydramine 50 mg IV, and the protocol was successfully performed in the Day Hospital Unit, with no breakthrough reactions.

**Table 1:** First rapid desensitization with Ocrelizumab 300 mg using the 3-bag, 12-step protocol published elsewhere (9).

					Total mg per bag	Amount of bag infused (ml)	
Solution 1	250	ml of	0.012	mg/ml	3.000	9.25	
Solution 2	250	ml of	0.120	mg/ml	30.000	18.75	
Solution 3	250	ml of	1.191	mg/ml	297.639	250.00	
**Step**	**Solution**	**Rate (ml/h)**	**Time (min)**	**Volume infused per step (ml)**	**Dose administered with this step (mg)**	**Cumulative dose (mg)**	**Fold increase per step**
1	1	2.0	15	0.50	0.0060	0.0060	–
2	1	5.0	15	1.25	0.0150	0.0210	2.5
3	1	10.0	15	2.50	0.0300	0.0510	2
4	1	20.0	15	5.00	0.0600	0.1110	2
5	2	5.0	15	1.25	0.1500	0.2610	2.5
6	2	10.0	15	2.50	0.3000	0.5610	2
7	2	20.0	15	5.00	0.6000	1.1610	2
8	2	40.0	15	10.00	1.2000	2.3610	2
9	3	10.0	15	2.50	2.9764	5.3374	2.48032
10	3	20.0	15	5.00	5.9528	11.2902	2
11	3	40.0	15	10.00	11.9056	23.1957	2
12	3	80.0	174.375	232.50	276.8043	300.0000	2
		Total time (minutes) = 339.375 = 5.66 h					

The total volume and dose dispensed are more than the final dose given to the patient because the initial solutions are not entirely infused.

Six months later, the patient received the next infusion, with a total 600 mg Ocrelizumab dose, under an RDD 12-step protocol, with no adverse reactions. However, the regular protocol for the injection of 600 mg indicates that the drug must be diluted in a 500-ml bag of saline solution. Thus, we adapted the RDD protocol so that the third bag (steps 9–12) included 500 ml. Still, infusion rates administered at each stage were doubled compared to the first desensitization (**Supplementary File**).

The patient signed the consent form, and the Ethics Committee from the University of São Paulo Medical School approved the study (CAAE 38855420.0.0000.0068, Plataforma Brasil).

## Discussion

We presented a female patient successfully desensitized to Ocrelizumab after an initial IHR. Although the reaction had not been severe, as the patient developed urticaria and angioedema, which is highly suggestive of mast cell activation despite the mechanisms involved, future infusions could induce anaphylaxis and be life threatening. On the other hand, the replacement of the MS therapy could lead to disease exacerbation. As far as we know, this is the first case published as a complete article in a journal. In 2019, two case reports were presented as abstracts in a scientific meeting (11).

It has been recently demonstrated that clinically significant IRRs include four major phenotypes: type-I-like hypersensitivity (IgE mediated or non-IgE mediated), cytokine-release, mixed reactions, and delayed type IV (12). A minority of individuals also present a type-III hypersensitivity reaction after biologic administration. It can include systemic serum-sickness disease or only a local Arthus reaction because of IgM and IgG deposition (12).

IRRs and cytokine-release reactions to mAbs can occur at first infusion and may typically present with mild to severe symptoms, including flushing, chills, fever, tachycardia, hypertension, dyspnea, nausea, vomiting, and syncope. The difference between IRRs and cytokine-release reactions is the self-limiting nature of IRRs on repeat exposure and the response to premedication (13).

Type I-like reactions to biologics can manifest with flushing, pruritus, urticaria, shortness of breath, hypotension, and life-threatening anaphylaxis that typically initiate during the infusion. These symptoms are associated with releasing mast cells/basophils mediators, including tryptase, histamine, leukotrienes, and prostaglandins, whose actions affect cutaneous, respiratory, gastrointestinal, and cardiovascular organ systems (12). They have delayed reactions that usually occur more than 12 h after the infusion and may range from mild maculopapular rash to severe cutaneous adverse reactions, such as Stevens–Johnson syndrome or drug rash with eosinophilia and systemic symptoms (12). Type-IV hypersensitivity includes <5% of mAb-induced responses.

Our female patient presented an immediate reaction characterized by flushing, followed by urticaria and facial angioedema, without respiratory or cardiovascular compromise. According to this classification, its phenotype could be considered a type-I-like hypersensitivity reaction based on clinical features. Type-III (14) and type-IV reactions (15, 16) have been previously associated with Ocrelizumab infusion, but type-I-like reactions have only been cited during phase III clinical trials (7).

Using a different classification of IRR severity that combined all kinds of adverse reactions, Mayer et al. described that more than 30% of Ocrelizumab-exposed individuals presented any IRR during clinical trials. It is essential to point out that all patients received a 100-mg dose of IV methylprednisolone before Ocrelizumab infusion (7). Our patient was also premedicated with methylprednisolone, which did not prevent the reaction. Considering the three trials altogether, more than 2,000 individuals were evaluated. Only one patient presented a severe Common Terminology Criteria for Adverse Events (CTCAE) grade IV IRR, namely, severe bronchospasm, during their first infusion in one of the OPERA studies. This treatment was then withdrawn (7). That severe reaction could have been a type-I-like hypersensitivity reaction. On the other hand, in ORATORIO, two patients presented severe IRRs, but the authors described clinical pictures as compatible with cytokine-release or mixed reactions, not a type-I-like reaction (7).

If we consider the Brown classification for severity of IHRs, our patient presented a grade I reaction (10). It is impossible to know if the reaction was less severe because of corticosteroid administration before the biologic. Thus, even if it was not severe, it probably involved mast cells and/or basophils activation, which could induce a future anaphylactic reaction during subsequent exposure. The patient became afraid of a future infusion, and the neurologist was concerned about her first-line therapy maintenance. Thus, we decided to perform an RDD with Ocrelizumab using the 12-step protocol published by Prof. Mariana Castells et al. (9, 17).

Although considered safe, rapid desensitization to Ocrelizumab has not been previously described. In the most extensive case series already published, including patients submitted to chemotherapeutic agents and biologics rapid desensitization, <30% of patients present any breakthrough reaction during the procedure. Moreover, <10% present severe breakthrough reactions, confirming that RDD is safe and effective (18, 19).

Mechanisms involved in Ocrelizumab hypersensitivity are unknown. Despite being clinically compatible with a type-I allergic reaction, we could not prove the involvement of IgE. IHRs induced by rituximab, another CD20-targeted mAb largely used in clinical practice to manage autoimmune diseases and hematological malignancies, can be IgE or non-IgE mediated (12). Our patient presented the IHR during the first infusion, but as we did not perform a skin test, it is impossible to postulate whether the reaction should be considered allergic or non-allergic. However, independently of the pathophysiology, RDD can be successfully performed in all type-I-like reactions, allowing maintenance of first-line therapy (12).

Our case description has a few limitations. First, we did not repeat standard infusion with a different premedication scheme, including an antihistamine. Nevertheless, it is also unlikely to be safe, since a 100 mg dose of IV methylprednisolone before infusion could not prevent the IHR. Furthermore, as cited above, we did not perform skin tests, making it impossible to define the mechanisms involved in the initial reaction. Future studies involving more individuals will permit a conclusion about the pathophysiology of hypersensitivity to Ocrelizumab.

In summary, we described that rapid desensitization with Ocrelizumab using the 3-bag, 12-step protocol is safe. It allowed the patient to be treated with the only approved disease-modifying therapy for PPMS and then prevent the progression of this severe neurological condition. Some other drugs, such as Ofatumumab and Rituximab, have been used off-label in selected cases. However, as they are still not approved for PPMS management, desensitization to Ocrelizumab can be safely considered to keep patients with this approved and efficacious treatment.

## Data Availability Statement

The original contributions presented in the study are included in the article/**Supplementary Material**. Further inquiries can be directed to the corresponding author.

## Ethics Statement

The study was reviewed and approved by University of São Paulo Medical School (CAAE 38855420.0.0000.0068, Plataforma Brasil). The patient provided her written informed consent to participate in this study. Written informed consent was obtained from the individual for the publication of any potentially identifiable images or data included in this article.

## Author Contributions

MA, FF, and VM assisted the patient and collected the data. MA, FF, and PG-B coordinated and performed the desensitization. MA and FF reviewed literature data. All authors contributed to the article and approved the submitted version.

## Conflict of Interest

The authors declare that the research was conducted in the absence of any commercial or financial relationships that could be construed as a potential conflict of interest.

## Publisher’s Note

All claims expressed in this article are solely those of the authors and do not necessarily represent those of their affiliated organizations, or those of the publisher, the editors and the reviewers. Any product that may be evaluated in this article, or claim that may be made by its manufacturer, is not guaranteed or endorsed by the publisher.
